# Which interventions increase hearing protection behaviors during noisy recreational activities? A systematic review

**DOI:** 10.1186/s12889-020-09414-w

**Published:** 2020-09-13

**Authors:** Michael T. Loughran, Stephanie Lyons, Christopher J. Plack, Christopher J. Armitage

**Affiliations:** 1 Manchester Centre for Health Psychology, School of Health Sciences, University of Manchester, Manchester Academic Health Science Centre, M13 9PL, Manchester, UK; 2 Manchester Centre for Audiology and Deafness, School of Health Sciences, University of Manchester, Manchester Academic Health Science Centre, Manchester, UK; 3 Department of Psychology, Lancaster University, Lancaster, UK; 4 Manchester University NHS Foundation Trust, Manchester Academic Health Science Centre, Manchester, UK

**Keywords:** Hearing protection intervention, Hearing conservation, Hearing protection behavior, Behavior change, Recreational noise-induced hearing loss, Recreational noise activity, Systematic review

## Abstract

**Background:**

Hearing loss and tinnitus are global concerns that can be reduced through hearing protection behaviors (e.g., earplug use). Little is known about the effectiveness of interventions to increase hearing protection use in recreational domains. For the first time we review systematically the effectiveness of such interventions.

**Methods:**

Systematic searches of nine databases, as well as grey literature and hand-searching, were conducted. Any study design was included if it assessed quantitatively a purposeful attempt to increase hearing protection in recreational settings. Studies were excluded if they assessed noise exposure from occupational sources and headphones/earphones, as these have been reviewed elsewhere. PROSPERO protocol: CRD42018098573.

**Results:**

Eight studies were retrieved following the screening of 1908 articles. Two pretest-posttest studies detected a small to medium effect (*d* ≥ 0·3 ≤ 0·5), one a small effect (*d* ~ =0·2) and two no real effect. Three posttest experimental studies detected small to medium effects (*d* ≥ 0·3 ≤ 0·5). Studies were rated as “poor quality” and 17 out of a possible 93 behavior change techniques were coded, with the majority targeting the intervention function ‘education’.

**Conclusions:**

Hearing loss and tinnitus due to recreational noise exposure are major public health concerns yet very few studies have examined preventive interventions. The present systematic review sets the agenda for the future development and testing of evidence-based interventions designed to prevent future hearing loss and tinnitus caused by noise in recreational settings, by recommending systematic approaches to intervention design, and implementation of intervention functions beyond education, such as incentivization, enablement and modeling.

## Background

Approximately one billion teenagers and young adults (12–35 years) are at risk of noise-induced hearing loss and tinnitus due to hazardous recreational noise exposure [1]. Recreational activities such as attendance at live music venues (nightclubs, festivals, concerts and bars), practising/producing music, do-it-yourself (DIY), engine noise and sports related noise [2–6] contribute the majority of risk, [7] with noise levels ranging between 91.7–140 dBA (A-weighted decibel), [8–12] and depending on duration of exposure all have the potential to cause hearing symptoms in a short space of time [13, 14]. Individuals who partake in recreational activities are more likely to have hearing loss than those who do not, [15] with dullness in hearing and tinnitus reported in up to 80% of people post activity [16, 17]. Recreational noise exposure can be reduced through the adoption of hearing protection behaviors, such as the use of hearing protection devices (earplugs and earmuffs) and regeneration breaks [18, 19]. However, people engaging in hearing protection behaviors during noisy recreational activities has been reported as fewer than 5%, [20, 21] and it is not known whether interventions have exerted measurable effect sizes post intervention in changing behaviors.

Previous hearing protection systematic and narrative reviews have investigated occupational settings, [22] recreational noise through personal listening devices that use headphones and earphones, [23] and education about hearing protection [24]. El Dib et al. [22] conducted a systematic review of randomised controlled trials in occupational settings designed to promote the wearing of earplugs and earmuffs and concluded that specifically tailored or individual based education interventions improved use of hearing protection. Diviani et al’s [23] narrative systematic review of personal listening devices identified two interventions [25, 26] indicating that warning signs and evocative imagery reduced volume levels. Kahn et al’s [24] systematic review focused on health education programmes targeted at youth and young adults’ use of hearing protection in occupational and recreational settings, identifying 10 studies that showed little evidence of effectiveness. However, given that education is just one out of nine possible functions that an intervention might serve (see Michie et al. [27]), it would be valuable to examine the effects of other intervention functions, such as environmental restructuring, modelling and incentivization as potential means to bring about health protection behavior change among people of all ages.

The use of health psychology theories and models have been discussed as a means to improving hearing health behavior change interventions, [28] with Coulson et al. [29] suggesting the ‘behavior change wheel’ framework and associated capability (C), opportunity (O) and motivation (M) model of behavior (B) change (COM-B) [27] as a new approach to use within this domain. The COM-B model is at the core of the framework, with capability, opportunity and motivation representative of the processes involved in enacting a behavior [27]. The COM-B model allows intervention designers to assess which drivers of the target behavior need to change. Once this has been established, then the remaining steps of the behavior change wheel framework help refine the components required for the target intervention, including intervention functions (categories of intervention), the behavior change techniques (active ingredients of the intervention), and the mode of delivering the final intervention [27].

The 93 techniques in the ‘Behavior Change Technique Taxonomy Version 1’ (BCTTv1) [30] are the smallest active ingredients of interventions and act as the catalysts energizing the appropriately identified intervention functions during the design process of the behavior change wheel [27]. For example, the technique ‘demonstration of the behavior (BCTTv1:6.1)’ would serve the intervention functions of both education and modelling. In previous preventative health behavior systematic reviews (e.g., physical activity) commonly used behavior change techniques including ‘goal setting (behavior) (BCTTv1:1.1)’, [31] and ‘feedback on behavior (BCTTv1:2.2)’ have been coded [32]. The coding of the behavior change techniques within hearing protection interventions will aid future intervention designers during this theory driven process. However, none of the previous hearing protection systematic [22, 24] and narrative reviews [23] have coded interventions to identify the behavior change techniques implemented according to the BCTTv1 taxonomy, [30] alongside extracting measurable hearing protection use outcome effects (Cohen’s d).

The present systematic review looks beyond occupational settings, includes hearing protection behaviors beyond personal listening device use, considers intervention functions in addition to education (e.g., incentivization [27]), and codes behavior change techniques for the first time in recreational hearing protection interventions. The aim of the present research is therefore to review systematically the literature on interventions designed to increase hearing protection behaviors in recreational settings.

The primary objectives of the current review are to: 1) quantify the effectiveness of hearing protection interventions in recreational noise domains, and 2) identify the active ingredients (behavior change techniques) of such interventions to help future designers of interventions to increase uptake and use of hearing protection behaviors in recreational settings.

## Methods

### Search strategy and selection criteria

This is a systematic review that followed the ‘Preferred Reporting Items for Systematic Reviews and Meta-Analyses’ guidance, [33] which was pre-registered on PROSPERO [CRD42018098573] on 6th June 2018. No meta-analysis could take place due to study heterogeneity. https://www.crd.york.ac.uk/prospero/display_record.php?RecordID=98573.

Searches were carried out on electronic databases: Cochrane Central Register of Controlled Trials; PubMED; EMBASE; MEDLINE; PsycINFO; Web of Science Core Collection; ComDisDome; Database of Abstracts of Reviews of Effects and Centre for Reviews and Dissemination. Grey literature was searched via: Grey Literature Report, Prospero, Open Grey, ClinicalTrials.gov, International Clinical Trials Registry. Hand-searching was conducted using the reference lists of papers that were included for full review. Authors of 12 studies were contacted for further clarification before inclusion or exclusion could be determined. The most recent full search was performed on 1st May 2020. All age groups, years and languages were considered for review.

A broad search strategy was developed alongside a research librarian to capture the large variety of hearing protection interventions (hearing conservation programs, education programs, hearing protection device use and noise legislation adherence). Keywords and structure to each search were altered depending on each electronic database (see Additional file 1); each strategy followed components related to intervention types, effects of noise exposure, hearing protection and different sound sources. Medical Subject Heading (MeSH) terms in this instance were not used due to the close association between occupational and recreational noise.

Studies were included if they assessed quantitatively a deliberate attempt to increase hearing protection behaviors when people are exposed to noise during recreational activities; study designs included ‘experimental posttest designs’ (randomized controlled trials and quasi-experimental studies) and ‘single group pretest-posttest designs’ (observational studies and surveys). Secondary outcomes included hearing health outcomes such as hearing loss or tinnitus as well as perceptions of capabilities, opportunities and motivations to engage in hearing protective behaviors. Interventions were also coded for the presence of the active ingredients aimed at delivering the desired change, namely, the behavior change techniques. The 93 techniques are clustered into 16 groups (e.g., goals and planning, reward and threat) within the behavior change technique taxonomy (BCTTv1), each technique has its own identifiable number for coding purposes, with the first digit identifying the cluster group, and the second the order of the technique within said group (e.g., habit formation: BCTTv1:8.1) [30]. These techniques were required to be observable, replicable, irreducible and to include a postulated active ingredient of the intervention. They were also required to be clearly defined to the target population(s) and target behavior(s)/outcome(s) within study methodologies [27, 30]. Coding the included studies clarified any effects of the intervention on primary and secondary outcomes. Studies were excluded if they assessed noise exposure from occupational sources and noise from personal listening devices while using headphones and/or earphones, as these have been reviewed elsewhere [22, 23].

The screening of abstracts and titles commenced with the 1st reviewer (ML) screening all articles and 2nd reviewer (SL) screening 10% of these reports for comparison. The 1st reviewer (ML) read all the fully eligible studies, with the 2nd and 3rd reviewers (SL and CJA) reading a proportion of the eligible studies, as well as discussing any query papers for full inclusion. Several authors were contacted directly to clarify information, leading to both inclusion and exclusion of studies for full review.

### Data extraction and analysis

**Fig. 1:**
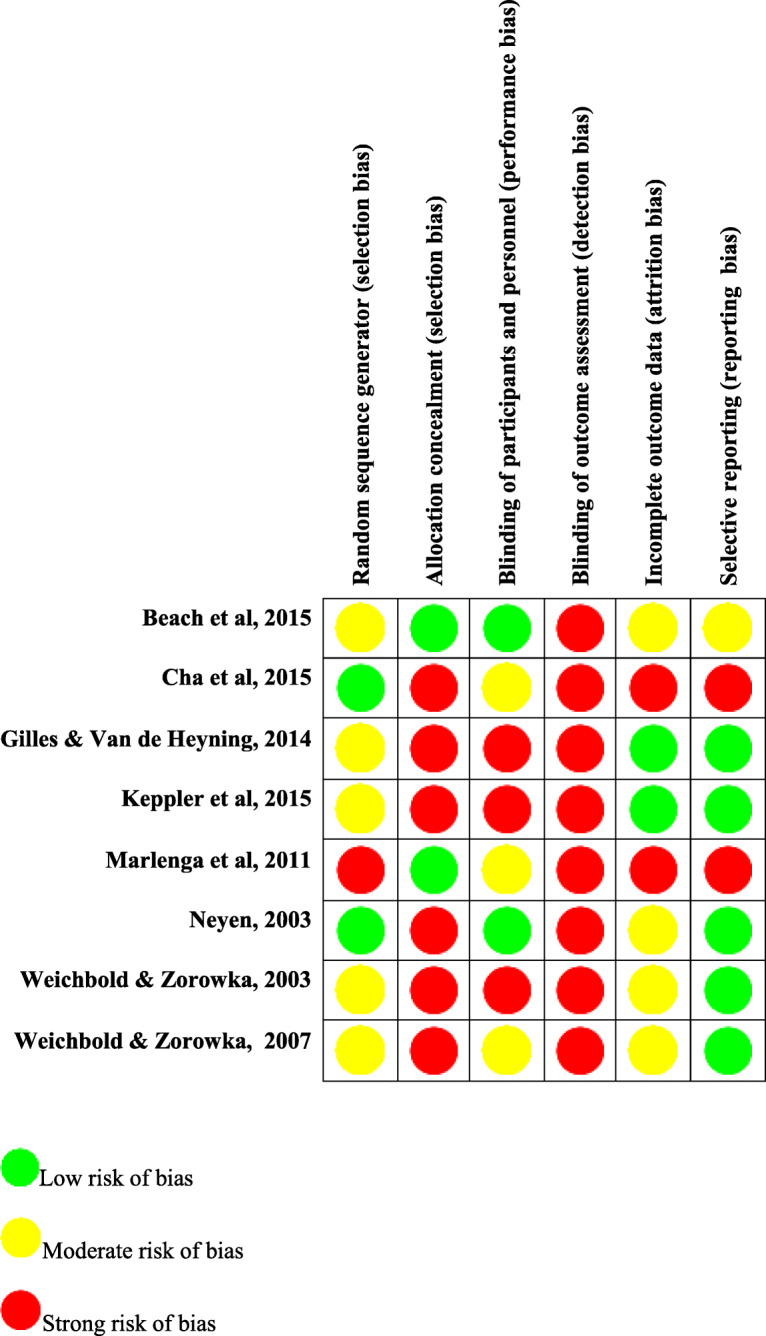
Cochrane Risk of Bias Table

Data were extracted using a pre-designed and piloted data extraction form, including general study information, study characteristics, participant characteristics, intervention design (including behavior change techniques), and outcome measures (see Table 1 and Additional file 2). Risk of bias and quality assessment were carried out independently by the 1st and 2nd reviewers using the Cochrane Quality Assessment Tool for systematic reviews [42]. The published protocol stated that the review would use the critical appraisal skills program (CASP) [43, 44]. However, with the included study designs not conforming to CASP checklists, and high levels of heterogeneity meaning determining the level of evidence using Cochrane’s ‘Grading of Recommendations, Assessment, Development and Evaluations’ tool was not possible, it was more appropriate to use the Cochrane Quality Assessment Tool for systematic reviews (see Fig. 1).

**Table 1:** Inclusion table with effect estimates and behavior change techniques

***Paper and Study Design***	***Study Aims***	***Characteristics***	Behavior Change Techniques (BCTTv1)*	***Outcome measures***	***Effect estimate***
**Beach et al, 2016** [34] ***Australia*** Experimental post-test design	Examine whether the presentation of hearing health information would result in increased use of earplugs, or whether provision of earplugs alone would be sufficient to change behavior. Experimental group (high level information) vs control group (low level information)	Age range: 20–39 Median age: 26, Average age: 27.1 Initial recruitment: 14 females and 37 males	**3.2.** Social support (practical) **4.1.** Instruction on how to perform a behavior **5.1.** Information about health consequences **5.2.** Salience of consequences **6.1.** Demonstration of the behavior **9.1.** Credible source **10.1.** Material incentive (behavior) **10.2.** Material reward (behavior) **12.5.** Adding objects to the environment	**Main:** earplug use in music venues **Time point:** 16 week follow up **Earplug use:** **Control:** 85.7% **Experimental:** 94.4%	RR = 1.1 95% CI = 0.9–1.36 Z = 0.916 **Cohen’s** ***d*** **= 0.3** (small to medium effect)
**Cha et al, 2015** [35] ***Canada*** Experimental post-test design	To provide information at three rock concerts (150–300 capacity) advertising free orange foam earplugs (intervention). With comparison to three other concerts with no earplugs available (control). The study wanted to measure and compare prevalence of earplug use at baseline (control) and when earplugs were available (intervention).	No age provided 955 participants; 318 intervention group (218 males, 100 females) 637 control group (410 males, 227 females)	**7.1.** Prompts/cues **10.1.** Material incentive **12.5.** Adding objects to the environment	**Main:** earplug use at Rock and Roll concerts **Time point:** In real time during concerts **Earplug use:** **Control:** 1.3% **Intervention:** 8.2%	RR = 6.51 95% CI = 2.98–14.22 Z = 4.702 **Cohen’s** ***d*** **= 0.31** (small to medium effect)
**Gilles & Van de Heyning, 2014** [36] ***Belgium*** Single group pretest-posttest design	Governmental preventive campaign (PrevC) to help prevent hearing damage caused by noise exposure. It was promoted via various ways such as television and radio commercials, social network sites (Facebook/Twitter), posters and a website. The campaign wanted to make young people more aware of the risks of loud music and therefore increase the use of hearing protection in noisy environments.	547 school children aged between 14 and 18 years Mean age = 16.8	**5.1.** Information about health consequences	**Main:** hearing protection use in noisy recreational environments **Time point:** 6 months post intervention **Earplug use:** **Baseline:** 3.7% **Follow up:** 14.3%	RR = 3.9 95% CI = 2.42–6.28 Z = 5.594 **Cohen’s** ***d*** **= 0.34** (small to medium effect)
**Keppler et al, 2015** [37] ***Belgium*** Single group pretest-posttest design	The study aim was to evaluate the effect of a hearing education program, including: attitudes and beliefs toward noise, hearing loss, and hearing protection device use in young adults.	18 years - 30 years Median age = 21.01 68 females; 10 males	**2.6.** Biofeedback **4.1.** Instruction on how to perform a behavior **5.1.** Information about health consequences **5.3.** Information about social and environmental consequences **9.1.** Credible source **9.2.** Pros and cons	**Main:** hearing protection use during noisy recreational activities **Time point:** 6 months post intervention Answers on 5 point Likert scale; closer to 1 equals improvement	**Baseline:** mean score = 3.40 S.D. = 1.36 **Follow up:** mean score = 2.94 S.D. = 1.37 **Cohen’s** ***d*** **= 0.34** (small to medium effect) Baseline and follow up data supplied by authors
**Marlenga et al, 2011** [38] ***USA*** Experimental post-test design	This paper is the 16 year longitudinal follow up of the rural hearing conservation intervention that is also the basis of the Berg et al. (2009**) study. The aim was to assess if the prevalence of hearing loss was reduced and that the use of hearing protection was maintained over the 16 year period. Berg et al. (2009**) described the historic intervention in more detail and it is from this paper that the behavior change techniques were coded.	Intervention group: 200 total 74.3% Male Median age = 31.2 Control group: 192 total 61.9% Male Median age = 30.8	**5.1.** Information about health consequences **5.2.** Salience of consequences **6.1.** Demonstration of the behavior **8.1.** Behavioral practice/rehearsal **8.3.** Habit formation **9.1.** Credible source **12.5.** Adding objects to the environment	**Main:** Earplug use during all recreational activities and gunfire. Stereo volume control for personal stereos **Time Point:** 16 year follow up **Earplug use all recreational activities:** **Control:** 16.9% **Intervention:** 20.4% **Personal stereos:** **Control:** 60.1% **Intervention:** 62.3% **Earplug use gunfire:** **Control:** 41.6% **Intervention:** 56.2%	**All recreational activities:** RR = 1.15 95% CI = 0.75–1.78 Z = 0.62 **Cohen’s d = 0.07** (no effect) **Personal stereos:** RR = 1.03 95% CI = 0.81–1.30 Z = 0.23 **Cohen’s d = 0.03** (no effect) **Gunfire:** RR = 1.37 95% CI = 1.04–1.80 Z = 2.23 **Cohen’s d = 0.3** (small to medium effect)
**Neyen, 2003** [39] ***Germany*** Single group pretest-posttest design	To assess music listening habits using questionnaires before and after a teaching unit; “hearing damage caused by loud music”. The study wanted to assess the extent of the transfer of knowledge and if there are any changes in awareness and behavior, including use of hearing protection at loud music events.	1674 participants in study; 873 male; 801 female	**5.1.** Information about health consequences **5.2.** Salience of consequences **9.3.** Comparative imaging of future outcomes	**Main:** hearing protection use at loud events **Time point:** 5/6 weeks post teaching session **Hearing protection use:** **Baseline:** 14.8% **Follow up:** 19.8%	RR = 1.34 95% CI = 1.41–1.58 Z = 3.614 **Cohen’s** ***d*** **= 0.14** (no effect)
**Weichbold & Zorowka, 2003** [40] ***Austria*** Single group pretest-posttest design	To measure the effects of a hearing campaign on the frequency of attendance of high school children at discotheques and whether they used hearing protection.	Baseline: 54 male and 115 female (169) Post intervention: 34 male and 93 female (136) Pre mean age = 16.9 Post mean age = 17.9	**4.1.** Instruction on how to perform a behavior **5.1.** Information about health consequences **5.2.** Salience of consequences **6.1.** Demonstration of the behavior	**Main:** earplug use at discotheques **Time point:** 1 year post intervention **Earplug use:** **Baseline:** 0% **Follow up:** 3.8%	RR = 14.17 95% CI = 0.8–253.9 Z = 1.80 **Cohen’s** ***d*** **= 0.21** (small effect)
**Weichbold & Zorowka, 2007** [41] ***Austria*** Single group pretest-posttest design	The study aims were the same as the previous study (Weichbold & Zorowka, 2003) with additional target behavior on taking regeneration breaks when exposed to noise at music events.	1757 participants at baseline 1535 at follow up Age pre-campaign: 16.2 +/− 1.3 years. Age post campaign: 17.0 +/− 1.2 years.	**4.1.** Instruction on how to perform a behavior **5.1.** Information about health consequences **6.1.** Demonstration of the behavior **8.1.** Behavioral practice/rehearsal **9.1.** Credible source **12.1.** Restructuring the physical environment	**Main:** earplug use and taking regeneration breaks at discotheques **Time point:** 1 year post intervention **Earplug use:** **Baseline:** 3.5% **Follow up:** 6.5% **Regeneration breaks:** **Baseline:** 89.9% **Follow up:** 91.7%	**Hearing protection:** RR = 1.84 95% CI = 1.36–2.52 Z = 3.885 **Cohen’s** ***d*** **= 0.14** (no effect) **Regeneration breaks:** RR = 1.02 95% CI = 1–1.04 Z = 1.772 **Cohen’s d = 0.06** (no effect)

* The behavior change techniques are coded according to the reference numbers provided within the behavior change technique taxonomy version 1, (BCTTv1) with the first digit associated to one of the 16 groups of clusters, and the second the order of the technique within said group (e.g., habit formation: BCTTv1:8.1). Michie S, Richardson M, Johnston M, Abraham C, Francis J, Hardeman W, Eccles MP, Cane J, Wood CE. The behavior change technique taxonomy (v1) of 93 hierarchically clustered techniques: building an international consensus for the reporting of behavior change interventions. Annals of behavioral medicine. 2013 Mar 20;46 (1):81–95

** Berg RL, Pickett W, Fitz-Randolph M, Broste SK, Knobloch MJ, Wood DJ, Kirkhorn SR, Linneman JG, Marlenga B. Hearing conservation program for agricultural students: short-term outcomes from a cluster-randomized trial with planned long-term follow-up. Preventive medicine. 2009 Dec 1;49 (6):546–52

Outcome measures were assessed in numerous different formats (often with multiple categories) and so the main outcome was recoded as ‘never-performers’ (never performed a hearing protection behavior) and ‘ever-performers’ (performed a hearing protection behavior at least some of the time). Quantitative data were extracted by the 1st and 2nd reviewer, and compared for agreement. The 3rd reviewer checked over the extracted quantitative data for agreement and calculation errors. One paper written in German was translated and included in the analysis [39]. Where studies included multiple time points, data were extracted from the last follow-up.

For dichotomous data, risk ratios and Z statistics were extracted; Cohen’s *d* was calculated using Z statistic and sample size (N) [45]. In terms of risk ratios, “ever-performers” of hearing protection behaviors were coded ‘positive outcomes,’ and “never-performers” were coded ‘negative outcomes.’ The cumulative incidence of the intervention group (or posttest data) was then divided by the cumulative incidence of the control group (or pretest data) (see Table 1). This approach is similar to that applied in the previous systematic review of occupational hearing protection behaviors [22]. One study presented data as adjusted means for the proportion of time hearing protection was used [38]; Cohen’s *d* could be inferred from the presented adjusted means and sample sizes provided [45].

The 1st and 2nd reviewers, trained via the University College London online behavior change technique taxonomy v1 program, [46] coded all included papers for behavior change techniques with an 80% agreement rate and a Cohen’s Kappa moderate agreement (κ = 0·58). The 3rd reviewer coded any disagreements and from this the final list of codes were agreed between all reviewers. Full coding of all behavior change techniques and associated taxonomy numbering can been found in Table 1*.*

## Results

**Fig. 2:**
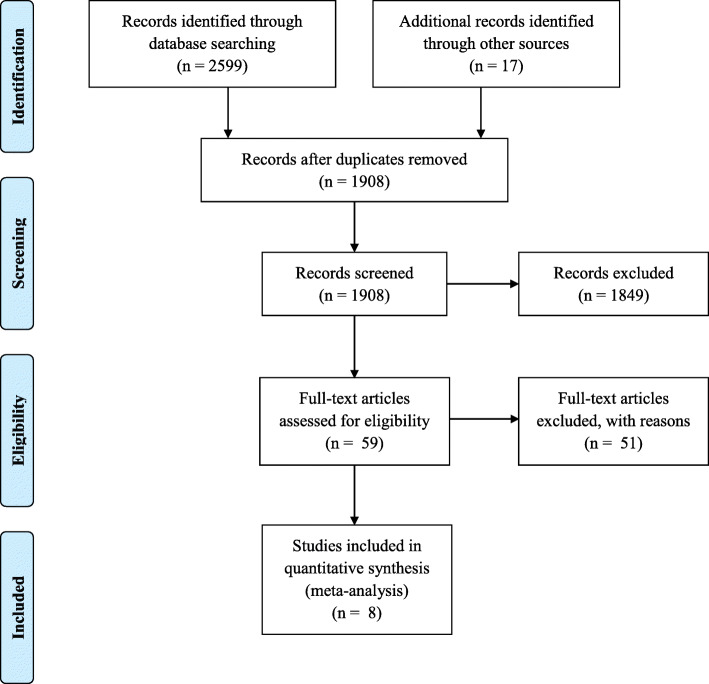
Preferred Reporting Items for Systematic Reviews and Meta-Analyses Flow Diagram

Initial searching recovered 2616 articles, of which 1908 (73%) were eligible for screening after the removal of duplicates (*n* = 708). Following screening, a total of 59 reports were eligible for full review, eight of which were suitable for inclusion according to our criteria (see Fig. 2). Five of these were single group pretest-posttest designs and three were experimental post-test designs (two randomized controlled trials and one quasi-randomized study). Fifty-one papers were excluded due to: studies missing or no behavioral data (*n* = 14); measurement of ‘intended behavior’ and not actual behavior (*n* = 13); no recognizable intervention (*n* = 11); occupational noise exposure (*n* = 6); unretrievable data (*n* = 5); acoustic intervention (*n* = 1); and contained historic data superseded by a subsequent paper (*n* = 1).

Individual effects were extracted for each study and are discussed in a narrative synthesis. Study heterogeneity occurred due to study design differences (five single group pretest-posttest designs and three experimental posttest designs), a range of follow up periods (16 weeks to 16 years) and a large population range (39 participants up to 1535 participants). All studies examined use of earplugs, one study additionally measured regeneration breaks alongside earplugs, [41] and a further study additionally measured adjustments of personal stereo volume through loudspeakers [38].

Seven of the included interventions were described as hearing conservation/education programs, [34, 36–41] with the final intervention focused solely on provision of free hearing protection devices (earplugs) versus no provision [35]. School children were the target of five studies, [36, 38–41] young adults (18–39 years) the target for two studies, [34, 37] and the final study did not define an age group or report an average age [35].

### Single pretest-posttest design studies

When examining the five single pretest-posttest designs we compared baseline data with final follow-up. Neyen’s [39] hearing conservation program delivered two hearing health teaching sessions, which included explanations of hearing loss (information about health consequences: BCTTv1: 5.1), audio simulations (salience of consequences: BCTTv1: 5.2) and experiments with volume levels (comparative imaging of future outcomes: BCTTv1: 9.3). The study detected only a very small increase in uptake of earplugs (*d* = 0·14) during loud events with German school children in the 5–6 weeks following the intervention. No secondary outcomes were reported. The study had high risk of allocation concealment bias as there was no randomization, and detection bias (blinding of outcomes) as blinding was not mentioned.

Weichbold and Zorowka’s [40] hearing conservation program “PROjectEAR” detected a small effect (*d* = 0·21) with increased use of earplugs at discotheques within Austrian high school children, 1 year post intervention. The children received four teaching sessions that included information on the risks of noise exposure (information about health consequences; BCTTv1: 5.1) alongside multimedia/listening examples (salience of consequences: BCTTv1: 5.2), and presentation of ear protection devices (instruction on how to perform a behavior: BCTTv1: 4.1; demonstration of the behavior: BCTTv1: 6.1). No secondary outcomes were reported. The study had high risk of allocation concealment bias, performance bias, and detection bias (blinding of outcomes), due to no randomization or mention of blinding.

Weichbold and Zorowka [41] continued with “PROjectEAR”, this time with a new and larger sample size of students (see Additional file 2) and an additional behavior measuring regeneration breaks. Additionally this version clearly stated the children received a talk from a hearing impaired person (credible source; BCTTv1: 9.1), with instruction to practice using earplugs (behavioral practice/rehearsal; BCTTv1: 8.1), and to remove themselves from noisy spaces (restructuring the physical environment; BCTTv1: 12.1); however, salience of consequences (BCTTv1: 5.2) was not explicitly coded this time around. The study detected a very small increase (*d* = 0·14) in earplug use 1 year post intervention, but no real effect (*d* = 0.06) of increased regeneration breaks. There are no reported secondary outcomes within this paper. Similarly, as within Weichbold and Zorowka [40] this study had high risk of allocation concealment bias and detection bias (blinding of outcomes).

Keppler et al.’s [37] hearing conservation program was performed by an audiologist (credible source; BCTTv1: 9.1) whom delivered feedback on hearing (biofeedback: BCTTv1: 2.6), educated on the risks of recreational noise, and discussed protective actions, including benefits/barriers (information about health consequences: BCTTv1: 5.1; information about social and environmental consequences: BCTTv1: 5.3: instruction on how to perform a behavior: BCTTv1: 4.1; pros and cons: BCTTv1: 9.2). The study detected a small to medium effect (*d* = 0·34) in increased use of hearing protection devices from baseline (mean 3·40; SD 1·36; range 1·00–5·00) to 6 months post intervention (mean 2·94; SD 1·37; range 1·00–5·00) within Belgian young adults. Secondary outcomes assessed the audiometric thresholds of the participants at baseline and 6 months post intervention, but no significant effects were found. Participant self-reports between sessions indicated that 28·2% of participants perceived their hearing loss to have increased, with 20·5% reporting that their tinnitus increased. However, the study had high risk of allocation concealment bias, performance bias, and detection bias, due to no randomization or mention of blinding.

The aim of Gilles and Van de Heyning’s [36] hearing conservation program was to make students aware of the dangers of loud music (information about health consequences: BCTTv1: 5.1) and therefore increase use of hearing protection; after administering questionnaires to Belgian students at baseline and 6 months post intervention, they detected a small to medium effect (*d* = 0·34) in increased use of devices while in noisy recreational environments. There are no reported secondary outcomes within this paper. The study was high risk of allocation concealment bias, performance bias, and detection bias, due to no randomization or mention of blinding.

### Experimental post-test designs

When examining the three experimental post-test designs we compared control and intervention data at the final follow-up. Marlenga et al. [38] completed a 16-year follow up of a hearing conservation program that was originally a clustered randomized controlled trial of rural American school children. Self-reported hearing protection use revealed a small to medium effect (*d* = 0·30) in the difference between groups for hearing protection use during gunfire. However, no effect was found for ‘all recreational activities’ (*d* = 0·07) or ‘personal stereos’ (*d* = 0·03). The program consisted of information on the ear and hearing delivered by a study educator, alongside videotape examples (credible source: BCTTv1: 9.1; information about health consequences; BCTTv1: 5.1; salience of consequences: BCTTv1: 5.2), and demonstrations/practice of how to fit hearing protection devices (demonstration of the behavior: BCTTv1: 6.1; behavioral practice/rehearsal: BCTTv1: 8.1); concluding with the provision of free devices that continued over the course of the intervention, alongside additional information (adding objects to the environment: BCTTv1: 12.5; habit formation: BCTTv1: 8.3). Secondary outcomes assessed changes in audiometric threshold frequencies from baseline across five categories, however no significant differences between groups were found for any of the categories. The study had high risk of detection bias as there is no mention of blinding, attrition bias due to dropouts, and reporting bias due to collection methods not known to be valid or reliable. There was also a high risk of selection bias due to a low agreement rate at the 16 year follow up, with an overall low risk of allocation concealment bias through initial randomization.

Beach et al.’s [34] hearing conservation program had the incentive of free earplugs and use demonstrated by an audiologist (material incentive (behavior): BCTTv1: 10.1; adding objects to the environment: BCTTv1: 12.5; credible source: BCTTv1: 9.1; instruction on how to perform a behavior: BCTTv1: 4.1; demonstration of the behavior: BCTTv1: 6.1), with a monetary reward for intervention completion (material reward (behavior): BCTTv1: 10.2). However, the experimental group received additional information on the dangers of noise, a video demonstrating hearing loss, and additional time with the audiologist (information about health consequences: BCTTv1: 5.1; salience of consequences: BCTTv1: 5.2; social support (practical): (BCTTv1: 3.2). The study detected a small to medium effect (*d* = 0·30) in differences of earplug use at live music events at 16 weeks post intervention within Australian young adults who regular attended live events. However, it is worth noting for this study that 82% of participants had used earplugs previously. This is unusually high and not representative of the general population, which is ∼5% for these types of events [21, 47]. Secondary outcomes were assessed with self-reporting of temporary thresholds shifts (76%) and tinnitus (92%) among all participants at follow up, with tinnitus reported as being permanent in 20% of the participants. The study reported as high risk of detection bias (blinding of outcome assessment) as it does not mention blinding of assessors, but with good internal validity due to randomization and blinding of participants.

When comparing control venues (three concerts) to experimental venues (three concerts) Cha et al. [35] detected a small to medium effect (*d* = 0·31) with greater use of earplugs being observed when earplugs were freely available at rock concerts (prompts and cues: BCTTv1: 7.1; material incentive (behavior): BCTTv1: 10.1). However, no secondary outcomes were measured meaning that it is not clear what was the mechanism of action. We considered this to be a quasi-study as it did not randomize participants but instead had control/interventions groups defined by venues, and was therefore high risk of allocation concealment bias; with large discrepancies in sample size between comparison groups. The study also had high risk of detection bias, attrition bias, and reporting bias, due to no mention of blinding or dropouts/withdrawals, and collection methods not known to be valid or reliable.

## Discussion

This review set out to assess, the effectiveness of recreational hearing protection interventions, and for the first time, to identify the active ingredients of these interventions. Considering hearing symptoms through recreational noise exposure is a global concern that is highly preventable, [1] only eight studies were retrieved that evaluated changes in hearing protection behaviors post intervention; only three studies have been carried out within the last 5 years, [34, 35, 37] indicating hearing protection interventions are being chronically under researched. Included studies lacked quality overall, with only three experimental post-test designs (two randomized controlled trials and one quasi study). Methodologies and results were poorly reported making it difficult to extract data, resulting in 12 authors being contacted throughout the review process. Furthermore, the poor quality of reporting highlights how difficult it would be, if not impossible, to replicate many of the interventions to further test outcomes. There is a need for more high quality experimental studies; improvements are required in terms of design quality and reporting to address this large gap in knowledge. This review included three studies [36–38] that were included in a previous systematic review, [24] but differed in interpretation because effect sizes (Cohen’s *d*) and behavior change techniques were extracted. Throughout screening it was apparent that interventions tended to measure people’s intentions to protect their hearing, as opposed to their actual hearing protection behaviors, [48–52] with a total of 13 papers rejected on this basis. Intentions do not represent the true effect of an intervention as people fail to act on their intentions approximately 50% of the time, [53] and this is an area that needs to be addressed with future hearing protection interventions.

The most common hearing protection behavior reported in each study was the use of earplugs. This supports previous evidence that hearing protection devices are the most accessible preventive method against overexposure to recreational noise [18, 48, 54]. While examining the effectiveness of the studies five indicated a small to medium effect (Cohen’s *d*), measuring an increase, or difference, in earplug use across a range of recreational contexts. However, those five studies had strong risk of bias, particularly detection bias (blinding of outcome assessment), with poorly reported methodologies affecting replication credentials.

The overall increase in ‘ever-performers’ of earplugs across all eight studies ranged from 3% – 14·6%; few studies indicate people always or often use earplugs, indicating many people are at risk of hearing symptoms in a short space of time, [13, 14] when these activities often have noise levels recorded at over 100 dBA [10–12]. Beach et al. [34] was the outlier of the group with 90% of all participants ‘ever-performers’; however, 82% were ‘ever-performers’ pre-study, which was perhaps due to targeting regular gig goers with an incentive of free earplugs. The present systematic review also highlights that preventative behaviors such as regeneration breaks, keeping safe distances, lowering sound levels, and adherence to legislations are less frequently addressed and should be avenues for future research.

Unique to the present systematic review was the coding of behavior change techniques deployed within hearing protection interventions, with a total of 17 coded as involving behavior change from a possible 93 techniques, the majority of which link to the intervention function ‘education’ (information about health consequences: BCTTv1:5.1). With so few techniques being identified it leaves a large scope to deploy previously untested techniques to bring about changes in hearing protection behaviors. Coding did reveal other less frequently used intervention functions, such as ‘environmental restructuring’ (adding objects to the environment: BCTTv1:12.5), that yielded consistent effect sizes when implemented. This particular deployment provided the most reliable relationship throughout the review; it was seen in all three experimental post-test studies, which provided earplugs within the environment, and all three measured a small to medium effect, for at least one recreational context. ‘Environmental restructuring’ should thus be considered for future recreational hearing protection interventions. Based on coding it would also be valuable to try other approaches in conjunction with environmental restructuring, such as incentivization (material incentive (behavior): BCTTv1.10.1), enablement (prompts and cues: BCTTv1:7.1) and modeling (salience of consequences: BCTTv1:5.2).

Studies indicate a lack of theory applied during the design process, or at least a lack of a description of theory within the methodologies. These issues have been raised by health psychologists previously, in that researchers face an uphill battle to replicate interventions to further test outcomes, [30, 55] due in part to poor reporting. Although one included paper applied the theory of planned behavior to design the evaluation questionnaires, [36] none of the included studies explicitly describes the use of theory for intervention design. The importance of behavior change theory and models (e.g., COM-B model) in hearing healthcare has been noted in the past, [28, 29] but appears still to be lacking within recreational hearing protection interventions. Therefore we would suggest better quality and more robust studies, achieved through use of theory and evidence, which will help target specific behavior change techniques and intervention functions to be incorporated in an effort to raise effect sizes. This use of theory driven practice will address gaps in knowledge in terms of quality and reporting for future systematic reviews, and help aid replication of interventions.

Limitations of this review include the inability to retrieve all data from the authors who were contacted directly. The provision of results that were missing or unclear in the original publications may have enabled a meta-analysis to be performed.

## Conclusions

The present systematic review found very few hearing protection interventions addressing recreational noise exposure, a global hearing health concern. However, ‘environmental restructuring’ through the provision of earplugs (adding objects to the environment: BCTTv1:12.5), showed promise and might be considered a starting point for future interventions. Further hearing protection intervention studies should be conducted that employ randomized controlled designs, use systematic approaches to intervention development (e.g., the behavior change wheel [27]), consider intervention functions beyond education, such as incentivization (e.g., material incentive (behavior): BCTTv1.10.1), enablement (e.g., prompts and cues: BCTTv1:7.1) and modeling (e.g., salience of consequences: BCTTv1:5.2), and consider deploying previously unused behavior change techniques. Self-reported use of hearing protection has been widely used as a main outcome measure, but more objective assessments through observation or technological solutions would reduce the risk of reporting bias.

## Supplementary information

**Additional file 1.** Search Strategy Supplement.

**Additional file 2.** Supplement Table.

## Data Availability

The datasets used and/or analysed during the current study are available from the corresponding author on reasonable request.

## Abbreviations

BCTTv1: Behavior Change Technique Taxonomy Version 1
CASP: Critical Appraisal Skills Program
COM-B: Capability, Opportunity and Motivation model of Behavior
dBA: A-weighted Decibel
DIY: Do-it-yourself
MeSH: Medical Subject Heading
