# Supplementary material for: Which interventions increase hearing protection behaviors during noisy recreational activities? A systematic review
Source: BMC Public Health. 2020 Sep 13;20:1376. doi: 10.1186/s12889-020-09414-w (PMC7488782; doi:10.1186/s12889-020-09414-w)
Supplement: Supplementary file 1 — Additional file 1. Search Strategy Supplement. [file 12889_2020_9414_MOESM1_ESM.docx]

**Additional File 1 – Search Strategy Supplement**

**WEB OF SCIENCE**

| #1 | Noise OR music |
| --- | --- |
| #2 | reduction OR abatement OR diminishment OR elimination |
| #3 | "hearing loss prevention" OR "hearing conservation" OR "hearing surveillance" OR “hearing education” OR “hearing protection behaviour” OR “hearing protection behavior” OR “hearing protection intervention” |
| #4 | Live music legislation* OR live music regulation* OR noise legislation* OR noise regulation* |
| #5 | Behavio* OR behavio* change |
| #6 | "ear protective device" OR "ear protective devices" OR "hearing protective device" OR "hearing protective devices" OR "hearing protector" OR "hearing protectors" OR "hearing protection" OR "ear muffs" OR "ear plugs" OR "ear defenders" OR “earplugs” OR “earmuffs” |
| #7 | Recreation* OR leisure |
| #8 | hearing OR hearing protect* |
| #9 | concert OR festival OR nightclub OR discotheque OR bar OR pub |
| #10 | Leisure activities OR firearms OR firearm* OR motorcycles OR motorcycle* OR motorbike* OR motor sport* OR sporting event* OR lawn mower* OR leaf blower* OR power tool* OR DIY |
| #11 | “Hearing loss” OR “noise induced hearing loss” OR “recreational noise induced hearing loss” OR “tinnitus” OR “noise induced” OR “recreational noise induced hearing loss” OR “noise damage” OR “noise reduction” |
| #12 | Noise OR hearing OR hearing protect* |
| #13 | **#1 AND #2** |
| #14 | **#1 AND #7** |
| #15 | **#1 AND #8** |
| #16 | **#1 OR #8** |
| #17 | **#10 AND #12** |
| #18 | **#9 AND #16** |
| #19 | **#5 AND #16** |
| #20 | **#3 OR #4 OR #6 OR #13 OR # 19** |
| #21 | **#14 OR #17 OR #18** |
| #22 | **#11 AND #20 AND #21** |

**COMDISDOME**

| #1 | Noise OR music |
| --- | --- |
| #2 | reduction OR abatement OR diminishment OR elimination |
| #3 | "hearing loss prevention" OR "hearing conservation" OR "hearing surveillance" OR “hearing education” OR “hearing protection behaviour” OR “hearing protection behavior” OR “hearing protection intervention” |
| #4 | Live music legislation* OR live music regulation* OR noise legislation* OR noise regulation* |
| #5 | Behavio* OR behavio* change |
| #6 | "ear protective device" OR "ear protective devices" OR "hearing protective device" OR "hearing protective devices" OR "hearing protector" OR "hearing protectors" OR "hearing protection" OR "ear muffs" OR "ear plugs" OR "ear defenders" OR “earplugs” OR “earmuffs” |
| #7 | Recreation* OR leisure |
| #8 | hearing OR hearing protect* |
| #9 | concert OR festival OR nightclub OR discotheque OR bar OR pub |
| #10 | Leisure activities OR firearms OR firearm* OR motorcycles OR motorcycle* OR motorbike* OR motor sport* OR sporting event* OR lawn mower* OR leaf blower* OR power tool* OR DIY |
| #11 | “Hearing loss” OR “noise induced hearing loss” OR “recreational noise induced hearing loss” OR “tinnitus” OR “noise induced” OR “recreational noise induced hearing loss” OR “noise damage” OR “noise reduction” |
| #12 | Noise OR hearing OR hearing protect* |
| #13 | **#1 AND #2** |
| #14 | **#1 AND #7** |
| #15 | **#1 AND #8** |
| #16 | **#1 OR #8** |
| #17 | **#10 AND #12** |
| #18 | **#9 AND #16** |
| #19 | **#5 AND #16** |
| #20 | **#3 OR #4 OR #6 OR #13 OR # 19** |
| #21 | **#14 OR #17 OR #18** |
| #22 | **#11 AND #20 AND #21** |

**OVID: Embase, PsycINFO, Medline, DARE , CDSR, CENTRAL**

Noise OR music AND (reduction OR abatement OR diminishment OR elimination)

**OR**

hearing loss prevention OR hearing conservation OR hearing surveillance OR hearing education OR hearing protect*

**OR**

ear protect* OR ear muffs OR ear plugs OR ear defenders OR earplugs OR earmuffs

**OR**

Live music legislation* OR live music regulation* OR noise legislation* OR noise regulation*

**OR**

Behavio* change AND (noise OR music OR recreational OR leisure)

**AND**

Noise OR music AND (recreation* OR leisure)

**OR**

Music OR noise OR hearing OR hearing protect* AND (concert OR festival OR nightclub OR discotheque OR bar OR pub)

**OR**

Leisure activities OR firearms OR firearm* OR motorcycles OR motorcycle* OR motorbike* OR motor sport* OR sporting event* OR lawn mower* OR leaf blower* OR power tool* OR DIY AND (noise OR hearing OR hearing protect*)

**AND**

Hearing loss OR noise induced hearing loss OR recreational noise induced hearing loss OR tinnitus OR noise induced OR recreational noise induced hearing loss OR noise damage OR noise reduction

**PubMed**

Noise OR music AND (reduction OR abatement OR diminishment OR elimination)

**OR**

"hearing loss prevention" OR "hearing conservation" OR "hearing surveillance" OR "hearing education" OR "hearing protection behaviour" OR "hearing protection behavior" OR "hearing protection intervention"

**OR**

"ear protective device" OR "ear protective devices" OR "hearing protective device" OR "hearing protective devices" OR "hearing protector" OR "hearing protectors" OR "hearing protection" OR "ear muffs" OR "ear plugs" OR "ear defenders" OR "earplugs" OR "earmuffs"

**OR**

Live music legislation* OR live music regulation* OR noise legislation* OR noise regulation*

**OR**

Behavio* AND change AND (noise OR music OR recreational OR leisure)

**AND**

Noise OR music AND (recreation* OR leisure)

**OR**

Music OR noise OR hearing OR hearing protect* AND (concert OR festival OR nightclub OR discotheque OR bar OR pub)

**OR**

Leisure activities OR firearms OR firearm* OR motorcycles OR motorcycle* OR motorbike* OR motor sport* OR sporting event* OR lawn mower* OR leaf blower* OR power tool* OR DIY AND (noise OR hearing OR hearing protect*)

**AND**

Hearing loss OR noise induced hearing loss OR recreational noise induced hearing loss OR tinnitus OR noise induced OR recreational noise induced hearing loss OR noise damage OR noise reduction
