# Supplementary material for: Which interventions increase hearing protection behaviors during noisy recreational activities? A systematic review
Source: BMC Public Health. 2020 Sep 13;20:1376. doi: 10.1186/s12889-020-09414-w (PMC7488782; doi:10.1186/s12889-020-09414-w)
Supplement: Supplementary file 2 — Additional file 2. Supplement Table. [file 12889_2020_9414_MOESM2_ESM.docx]

***Additional File 2 - Supplement Table***

| ***Paper*** | ***Participant Results*** | ***Intervention Type*** | ***Recruitment*** |
| --- | --- | --- | --- |
| **Beach et al, 2016**  ***Australia***  Experimental post-test design | High information group: 18 total; 17 used earplugs (ever performers)  Low information group: 21 total; 18 used earplugs (ever performers) | Hearing conservation/education program.  All participants provided with free earplugs and shown how to use by an audiologist.  High information group received additional material: 3 minute video presentation, 2 page brochure, 2 additional emails of audio simulation and link to music focused noise reduction campaign | People who attend loud events.  Advertisements on music websites.  Contacted by telephone and a face to face interview arranged. 82% of those recruited said they had used earplugs in the past. |
| **Cha et al, (2015)**  ***Canada***  Experimental post-test design | 318 in intervention group; 26 wore earplugs (ever performers)  637 in control group; 8 wore earplugs (ever performers) | Provision of free earplugs  Earplug provision alongside signage of free earplugs being available (intervention)  No free available earplugs and no signage (control) | Two venues of similar size and capacity were chosen across six similar type concerts.  Recruitment depended upon the number of attendees that attended each concert. |
| **Gilles & Van de Heyning, (2014)**  ***Belgium***  Single group pretest-posttest design | 547 at baseline; 20 used earplugs (ever performers)  547 at follow up; 78 used earplugs (ever performers)  (data supplied by author) | Government advertising campaign  Intervention implemented through uses such as television and radio commercials, social  media sites (Facebook/Twitter), posters and a website.  Comparing before and after questionnaires within the cohort  No hearing protection devices provided | Schools were contacted by the research team and if the school agreed written communication was sent out, including the questionnaire. The principle of the school could then administer the questionnaires at baseline and post intervention. |
| **Keppler et al, 2015**  ***Belgium***  Single group pretest-posttest design | n = 78 pre and post  (means and standard deviation supplied by author) | Hearing conservation/education program.  Two sessions in which questionnaires administered. Hearing tests also completed at both sessions. Feedback on hearing was given after the first test. All then received an education teaching session from an audiologist on a one-one basis; discussing dangers of recreational noise and preventative measures.  No hearing protection devices provided | Unclear - states young adults participated voluntarily. |
| **Marlenga et al, 2011**  ***USA***  Experimental post-test design | Hearing protection reported as adjusted mean (%) of time used  **All recreational activities**  Control: n =163; mean =16.9%  Intervention: n =181; mean =20.4%  **Personal Stereos**  Control: n =93; mean =60.1%  Intervention: n =84; mean =62.3%  **Gunfire**  Control: n =104; mean =41.6%  Intervention: n =131; mean =56.2% | Hearing conservation/education program.  The original 3 year intervention included a classroom teaching session with the distribution of hearing protection. The intervention was reinforced by mailing the participants information at different time points throughout, as well as making hearing protection available at different time points. | 392 participants from the original study were recruited through searching medical records, telephone directories and internet searches. Efforts were then made to make contact and await replies. |
| **Neyen, 2003**  ***Germany***  Single group pretest-posttest design | 1565 at baseline; 231 had used earplugs (ever performers)  1362 at follow up; 270 had used earplugs (ever performers) | Hearing conservation/education program.  Involved a teaching unit about how loud music can cause hearing damage. A questionnaire was administered before and directly after the teaching session. A third was administered 5/6 weeks post session. The teaching lesson discussed the physics of sound/music, structure of hearing, symptoms of overexposure (hearing loss/tinnitus). The session was delivered by the differing school teachers during their normal classroom time. | Each school was approached and asked if they would like to participate, with consent letters sent out to parents; 14 schools recruited; totalling 92 classes  1565 participants answered hearing protection questions at baseline; 1362 at follow up |
| **Weichbold & Zorowka 2003**  ***Austria***  Single group pretest-posttest design | 169 at baseline; 0 used earplugs (ever performers)  131 at follow up; 5 used earplugs (ever performers) | Hearing conservation/education program  Prorgram called “PROjectEAR”. Consisted of four 45-minute sessions over three days which included: lectures, media presentations, group work, presentation of hearing protection devices/hearing aids, practical exercises and role-play. Participants received information on: function of ear, importance in life, adverse effects of hearing loss on everyday activities. Particularly damaged caused by continuous exposure to recreational noise.  No hearing protection devices provided | PROjectEAR campaign took place at 6 high schools |
| **Weichbold & Zorowka 2007**  ***Austria***  Single group pretest-posttest design | **Hearing protection:**  1757 at baseline; 62 used earplugs (ever performers)  1535 at follow up; 100 used earplugs (ever performers)  **Regeneration breaks:**  1757 at baseline; 1580 took breaks (ever performers)  1535 at follow up; 1407 took breaks (ever performers) | Intervention design remained the same as the 2003 study (PROjectEAR)  No hearing protection devices provided | Austrian schools that have the campaign in the curriculum since 2002. |
